# Methyl α-l-rhamnosyl-(1→2)[α-l-rhamnosyl-(1→3)]-α-l-rhamnoside penta­hydrate: synchrotron study

**DOI:** 10.1107/S1600536812027390

**Published:** 2012-06-27

**Authors:** Lars Eriksson, Göran Widmalm

**Affiliations:** a Department of Material and Environmental Chemistry, Arrhenius Laboratory, Stockholm University, SE-106 91 Stockholm, Sweden; b Department of Organic Chemistry, Arrhenius Laboratory, Stockholm University, SE-106 91 Stockholm, Sweden

## Abstract

The title hydrate, C_19_H_34_O_13_·5H_2_O, contains a vicinally disubstituted tris­accharide in which the two terminal rhamnosyl sugar groups are positioned adjacent to each other. The conformation of the tris­accharide is described by the glycosidic torsion angles ϕ2 = 48 (1)°, ψ2 = −29 (1)°, ϕ3 = 44 (1)° and ψ3 = 4 (1)°, whereas the ψ2 torsion angle represents a conformation from the major state in solution, the ψ3 torsion angle conformation may have been caught near a potential energy saddle-point when compared to its solution structure, in which at least two but probably three conformational states are populated. Extensive inter­molecular O—H⋯O hydrogen bonding is present in the crystal and a water-containing channel is formed along the *b*-axis direction.

## Related literature

For a description of l-rhamnose as part of polysaccharides, see: Marie *et al.* (1998 ▶); Perry & MacLean (2000 ▶). For a description of the conformational dynamics of the title tris­accharide, see: Eklund *et al.* (2005 ▶); Jonsson *et al.* (2011 ▶). For a description of the puckering analysis of the residues, see: Cremer & Pople (1975 ▶). For further background to l-rhamnose, see: Ansaruzzaman *et al.* (1996 ▶); Varki *et al.* (1999) ▶; Kulber-Kielb *et al.* (2007 ▶); Lindberg (1998 ▶); Säwén *et al.* (2010 ▶).
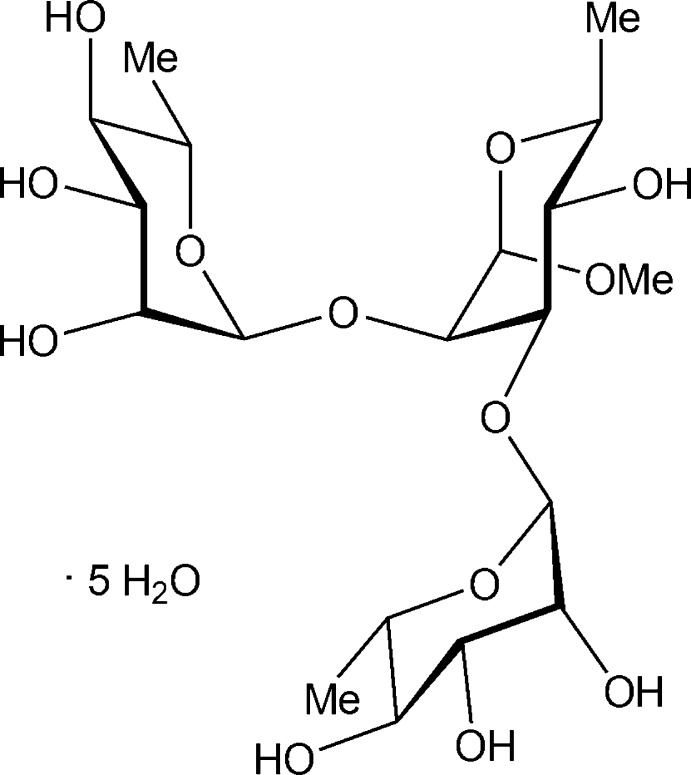


## Experimental

### 

#### Crystal data

C_19_H_34_O_13_·5H_2_O
*M* *_r_* = 560.54
Monoclinic, 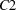

*a* = 19.345 (3) Å
*b* = 6.4870 (13) Å
*c* = 21.145 (3) Å
β = 97.617 (14)°
*V* = 2630.0 (8) Å^3^
*Z* = 4
Synchrotron radiation
λ = 0.8970 Å
μ = 0.22 mm^−1^
*T* = 100 K
0.20 × 0.05 × 0.01 mm

#### Data collection

Bruker SMART 1K CCD diffractometer
Absorption correction: multi-scan (*SADABS*; Sheldrick, 2002 ▶) *T*_min_ = 0.97, *T*_max_ = 0.99
17172 measured reflections
2906 independent reflections
2655 reflections with *I* > 2σ(*I*)
*R*_int_ = 0.046

#### Refinement

*R*[*F* ^2^ > 2σ(*F* ^2^)] = 0.033
*wR*(*F* ^2^) = 0.087
*S* = 1.07
2906 reflections
376 parameters
16 restraints
H atoms treated by a mixture of independent and constrained refinement
Δρ_max_ = 0.56 e Å^−3^
Δρ_min_ = −0.29 e Å^−3^

### 

Data collection: *SMART* (Bruker, 1997 ▶); cell refinement: *SAINT* (Bruker, 1997 ▶); data reduction: *SAINT*; program(s) used to solve structure: *SHELXS97* (Sheldrick, 2008 ▶); program(s) used to refine structure: *SHELXL97* (Sheldrick, 2008 ▶); molecular graphics: *DIAMOND* (Brandenburg, 1999 ▶); software used to prepare material for publication: *PLATON* (Spek, 2009 ▶).

## Supplementary Material

Crystal structure: contains datablock(s) I, global. DOI: 10.1107/S1600536812027390/hb6841sup1.cif

Structure factors: contains datablock(s) I. DOI: 10.1107/S1600536812027390/hb6841Isup2.hkl

Additional supplementary materials:  crystallographic information; 3D view; checkCIF report

## Figures and Tables

**Table 1:** Hydrogen-bond geometry (Å, °)

*D*—H⋯*A*	*D*—H	H⋯*A*	*D*⋯*A*	*D*—H⋯*A*
O*W*1—H101⋯O33^i^	0.88 (3)	1.85 (3)	2.726 (2)	176 (2)
O*W*1—H102⋯O*W*3^ii^	0.88 (3)	1.95 (3)	2.802 (2)	162 (2)
O*W*1—H102⋯O32^iii^	0.88 (3)	2.55 (3)	2.976 (2)	110 (2)
O*W*2—H201⋯O12^iv^	0.88 (3)	2.03 (3)	2.875 (2)	163 (2)
O*W*2—H202⋯O35^ii^	0.87 (3)	2.08 (3)	2.877 (2)	153 (2)
O*W*3—H301⋯O*W*5	0.88 (3)	2.04 (3)	2.845 (2)	151 (2)
O*W*3—H302⋯O13^v^	0.88 (3)	1.96 (3)	2.836 (2)	176 (2)
O*W*4—H401⋯O*W*3	0.88 (3)	1.97 (3)	2.840 (2)	168 (2)
O*W*4—H402⋯O*W*1	0.88 (3)	1.92 (3)	2.771 (2)	160 (2)
O*W*5—H501⋯O33^vi^	0.87 (3)	2.07 (3)	2.918 (2)	168 (2)
O*W*5—H502⋯O*W*5^vii^	0.87 (3)	2.50 (3)	3.333 (2)	159 (2)
O12—H12*A*⋯O32^iii^	0.84	2.01	2.767 (2)	149
O13—H13*A*⋯O15^ii^	0.84	2.10	2.858 (2)	149
O14—H14*A*⋯O24^iii^	0.84	1.95	2.733 (2)	157
O24—H24*A*⋯O*W*2	0.84	1.88	2.722 (2)	176
O32—H32*A*⋯O*W*5^viii^	0.84	2.13	2.864 (2)	146
O33—H33*A*⋯O34^i^	0.84	1.91	2.684 (2)	152
O34—H34*A*⋯O*W*4	0.84	1.86	2.687 (2)	168

Symmetry codes: (i) 
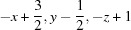
; (ii) 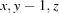; (iii) 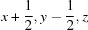; (iv) 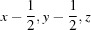; (v) 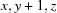; (vi) 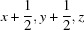; (vii) 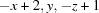; (viii) 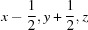.

**Fig. 1.**
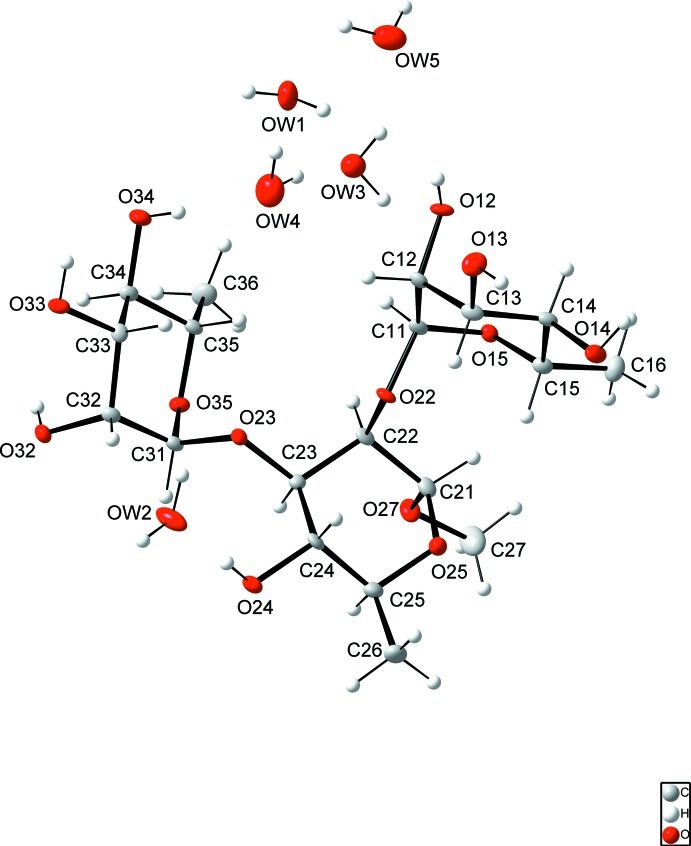
A view of the molecule with displacement ellipsoids drawn at the 50% probablity level.

**Fig. 2.**
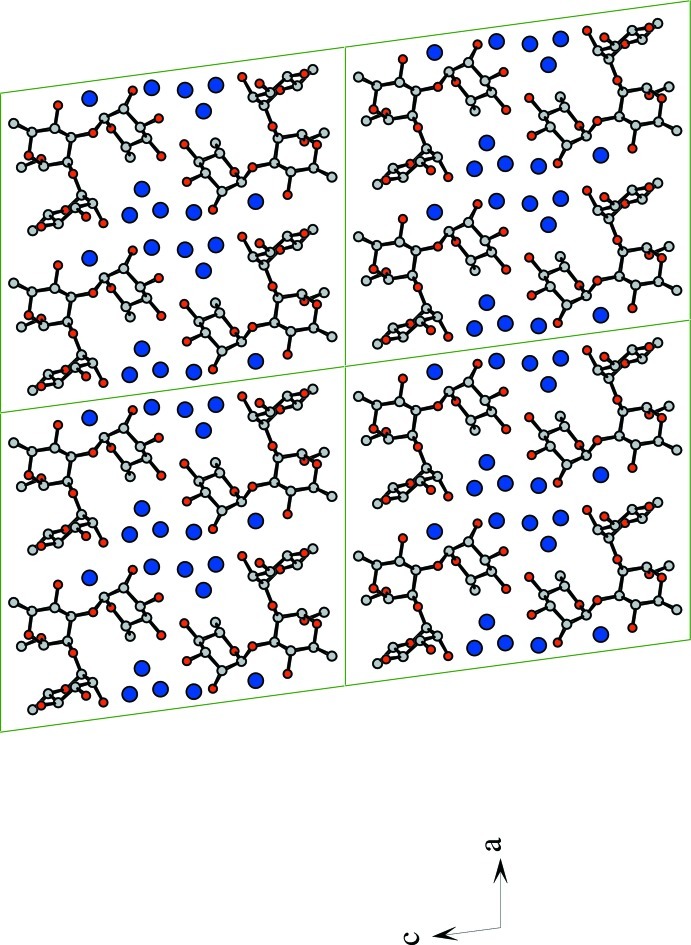
Four unit cells viewed along the b axis with the water molecules symbolized by the large blue discs. The water molecules mediate intermolecular hydrogen bonds between the sugar molecules and along the b axis.

**Fig. 3.**
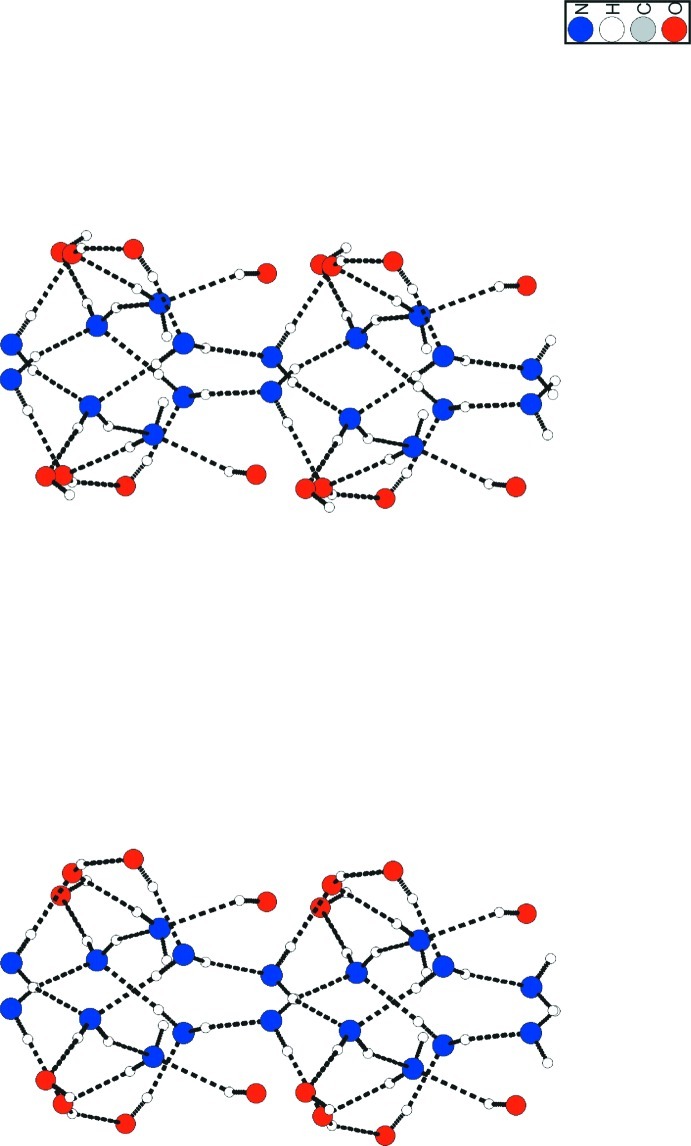
Stereoview of the hydrogen bonded water structure of approximately two unit-cell lengths along the b axis. The water O atoms are shown with blue color and the hydroxyl O atoms are shown with red color.

## supplementary crystallographic information

### Comment

In carbohydrate structures from humans the number of different monosaccharides is quite limited; typically seven different sugars are present in glycoproteins and glycolipids (Varki *et al.*, 1999). Constituents of polysaccharides in man add a few more monosaccharides to the repertoire. In bacteria, however, more than 100 different monosaccharide components have been found (Lindberg, 1998). One of them, *L*-rhamnose (6-deoxy-*L*-mannose) is present as a major constituent of the O-antigen polysaccharides from Shigella flexneri (Kulber-Kielb *et al.*, 2007) and is the sole monosaccharide in the repeating unit of an O-antigen from a Klebsiella pneumoniae strain (Ansaruzzaman *et al.*, 1996). *L*-rhamnose is also found a the branch point sugar in some polysaccharides, *e.g.*, from *Escherichia coli* O139 (Marie *et al.*, 1998) and Yersinia enterocolitica serotype O:28 (Perry & MacLean, 2000).

In the title compound (I) the three sugar components are all *L*-rhamnose residues having the α-anomeric configuration. The *O*-methyl residue (*a*) is vicinally disubstituted at O2 (residue b) and O3 (residue c) which leads to spatial proximity of also the two latter rhamnosyl groups. The major degrees of freedom in trisaccharide (I) are present at the (1 → 2)- and (1 → 3)-linkages, *i.e.*, between residues b and a as well as between residues c and a, respectively. The torsion angles are given by φ2 = 48°, ψ2 = -29°, φ3 = 44° and ψ3 = 4°. In a recent NMR and molecular dynamics (MD) simulation study of (I) in water solution <φ> ≈ 40°, when the *exo*-anomeric conformation was populated, but non-*exo* conformations with φ < 0° were also significantly populated (Eklund *et al.*, 2005). The dynamics of the ψ torsion angles were found to be highly correlated with both ψ2 and ψ3 being either > 0° or < 0°. The conformation of the X-ray structure (Figure 1) is reminiscent of the conformational states found from the MD simulation and the values of the glycosidic torsion angles are observed to correspond to conformational regions that are highly populated, albeit the ψ torsion angles in the solid state structure deviate somewhat from the pattern observed from the molecular simulations with water as a solvent.

In studies of the conformational dynamics of the title trisaccharide *trans*-glycosidic heteronuclear carbon-proton coupling constants were measured (Eklund *et al.*, 2005; Jonsson *et al.*, 2011) which, when interpreted by Karplus-type relationships (Säwén *et al.*, 2010), can yield information on conformation *via* torsion angles at the glycosidic linkages. Calculation of the three-bond coupling constants based on the torsion angles in the crystal structure of the trisaccharide showed that for the φ torsion angles and the ψ torsion angle at the α-(1 → 2)-linkage the differences to the experimental data were not larger than *ca* 0.5 Hz, indicating that for these torsions the conformation in the solid state is similar to that populated to a large extent in solution. However, for the ψ torsion angle at the α-(1 → 3)-linkage the corresponding difference was larger, *ca* 1 Hz, suggesting that in the crystal structure the latter torsion describes a conformation that is less populated in water solution. The crystal structure conformation is still, however, one in a low potential energy region, since conformational exchange occurs for both of the ψ torsion angles between states for which ψ takes either positive or negative values according to the molecular dynamics simulation (Eklund *et al.*, 2005).

The calculated Cremer & Pople (1975) parameters for the three different rings are: ring O15 → C15 [Q=0.570 (2) Å, θ=177.9 (2) ° and φ=20 (9) °], ring O25 → C25 [Q=0.580 (2) Å, θ=171.4 (2) ° and φ=72.5 (14) °] and for the ring O35 → C35 [Q=0.582 (2) Å, θ=177.1 (2) ° and φ=131 (5) °].

Extensive water-water hydrogen bonding was observed (Table 1) between the title compound and water molecules leading to a water channel in the b-direction (Fig. 2 and Fig. 3). The title compound showed hydrogen bonds to water and to other adjacent (symmetry related) trisaccharides, but no intra-molecular hydrogen bonds were found.

### Experimental

The synthesis of (I) was described by Eklund *et al.* (2005) in which all three rhamnosyl residues have the *L* absolute configuration. The trisaccharide was crystallized at ambient temperature by slow evaporation from a mixture of water and ethanol (1:1). The crystal was mounted in a capillary tube and diffraction data were collected at 100 K on beamline I711 at the Swedish synchrotron radiation facility, MAXLAB, Lund.

### Refinement

All hydrogen atoms, except those on the water molecules, were geometrically placed and constrained to ride on the parent atom. The C—H bond distances are 0.98 Å for CH_3_, 0.99 Å for CH_2_, 1.00 Å for CH. The O—H bond distance is 0.84 Å for OH groups. The *U*_iso_(H) = 1.5 *U*_eq_(C,*O*) for the CH_3_ and OH while it was set to 1.2 *U*_eq_(C) for all other H atoms. Due to the abscence of significant anomalous scatterers, the value of the Flack parameter was not meaningful, thus the 3220 Friedel equivalents were included in the merging process (MERG 4 in *SHELXL*). The absolute configuration of each sugar residue is known from the starting compounds used in the synthesis. The hydrogen atoms of the water molecule were located from difference density map, given *U*_iso_(H) = 1.5*U*_eq_(O) and in the refinement the d(O—H) and d(H..H) were restrained to retain the previously known geometry of the water molecule. The H502 is an hydrogen atom connected to a solvent water molecule where the H502 related by a 2 fold axis will be positioned at a much too close distance. The water molecule defined by OW5, H501 and H502 do not strictly fulfil the crystallographic symmetry of the rest of the strucutre, at least this is true for one of the H atoms for this very water molecule.

### Crystal data

**Table:**

C_19_H_34_O_13_·5H_2_O	*F*(000) = 1208
*M**_r_* = 560.54	*D*_x_ = 1.416 Mg m^−^^3^
Monoclinic, *C*2	Synchrotron radiation, λ = 0.8970 Å
Hall symbol: C 2y	Cell parameters from 963 reflections
*a* = 19.345 (3) Å	θ = 2.5–39.8°
*b* = 6.4870 (13) Å	µ = 0.22 mm^−^^1^
*c* = 21.145 (3) Å	*T* = 100 K
β = 97.617 (14)°	Plate, colourless
*V* = 2630.0 (8) Å^3^	0.20 × 0.05 × 0.01 mm
*Z* = 4	

### Data collection

**Table:**

Bruker SMART 1K CCD diffractometer	2906 independent reflections
Radiation source: Beamline I711, Maxlab	2655 reflections with *I* > 2σ(*I*)
Silicon monochromator	*R*_int_ = 0.046
Detector resolution: 10 pixels mm^-1^	θ_max_ = 34.1°, θ_min_ = 2.5°
ω scan at different φ	*h* = −23→24
Absorption correction: multi-scan (*SADABS*; Sheldrick, 2002)	*k* = −8→8
*T*_min_ = 0.97, *T*_max_ = 0.99	*l* = −23→26
17172 measured reflections	

### Refinement

**Table:**

Refinement on *F*^2^	Primary atom site location: structure-invariant direct methods
Least-squares matrix: full	Secondary atom site location: difference Fourier map
*R*[*F*^2^ > 2σ(*F*^2^)] = 0.033	Hydrogen site location: inferred from neighbouring sites
*wR*(*F*^2^) = 0.087	H atoms treated by a mixture of independent and constrained refinement
*S* = 1.07	*w* = 1/[σ^2^(*F*_o_^2^) + (0.0607*P*)^2^] where *P* = (*F*_o_^2^ + 2*F*_c_^2^)/3
2906 reflections	(Δ/σ)_max_ < 0.001
376 parameters	Δρ_max_ = 0.56 e Å^−^^3^
16 restraints	Δρ_min_ = −0.29 e Å^−^^3^

### Special details

**Table:**

Geometry. All e.s.d.'s (except the e.s.d. in the dihedral angle between two l.s. planes) are estimated using the full covariance matrix. The cell e.s.d.'s are taken into account individually in the estimation of e.s.d.'s in distances, angles and torsion angles; correlations between e.s.d.'s in cell parameters are only used when they are defined by crystal symmetry. An approximate (isotropic) treatment of cell e.s.d.'s is used for estimating e.s.d.'s involving l.s. planes.
Refinement. Refinement of *F*^2^ against ALL reflections. The weighted *R*-factor *wR* and goodness of fit *S* are based on *F*^2^, conventional *R*-factors *R* are based on *F*, with *F* set to zero for negative *F*^2^. The threshold expression of *F*^2^ > σ(*F*^2^) is used only for calculating *R*-factors(gt) *etc*. and is not relevant to the choice of reflections for refinement. *R*-factors based on *F*^2^ are statistically about twice as large as those based on *F*, and *R*- factors based on ALL data will be even larger.

### Fractional atomic coordinates and isotropic or equivalent isotropic displacement parameters (Å^2^)

**Table:**

	*x*	*y*	*z*	*U*_iso_*/*U*_eq_	
OW1	0.93266 (10)	−0.3863 (3)	0.46456 (9)	0.0236 (4)	
H101	0.9098 (15)	−0.456 (4)	0.4905 (12)	0.035*	
H102	0.9424 (17)	−0.474 (4)	0.4352 (12)	0.035*	
OW2	0.55372 (9)	−0.3755 (3)	0.25876 (10)	0.0244 (4)	
H201	0.5191 (11)	−0.458 (4)	0.2626 (15)	0.037*	
H202	0.5911 (10)	−0.422 (5)	0.2816 (14)	0.037*	
OW3	0.93523 (9)	0.2971 (3)	0.37476 (9)	0.0213 (4)	
H301	0.9740 (11)	0.225 (5)	0.3809 (13)	0.032*	
H302	0.9301 (15)	0.339 (5)	0.3350 (8)	0.032*	
OW4	0.85929 (11)	−0.0490 (3)	0.41056 (10)	0.0321 (5)	
H401	0.8879 (15)	0.052 (4)	0.4032 (17)	0.048*	
H402	0.8891 (14)	−0.133 (4)	0.4327 (16)	0.048*	
OW5	1.04589 (10)	0.0556 (3)	0.43863 (9)	0.0271 (4)	
H501	1.0791 (14)	0.144 (5)	0.4484 (14)	0.041*	
H502	1.0265 (15)	0.022 (5)	0.4722 (11)	0.041*	
C11	0.85217 (11)	−0.0103 (3)	0.23565 (11)	0.0109 (5)	
H11	0.8527	0.0985	0.2692	0.013*	
C12	0.88270 (11)	−0.2061 (3)	0.26740 (11)	0.0116 (5)	
H12	0.8528	−0.2573	0.2992	0.014*	
C13	0.88938 (12)	−0.3693 (3)	0.21708 (11)	0.0126 (5)	
H13	0.8419	−0.4074	0.1957	0.015*	
C14	0.93200 (12)	−0.2870 (3)	0.16795 (11)	0.0116 (5)	
H14	0.9806	−0.2587	0.1886	0.014*	
C15	0.89936 (12)	−0.0874 (4)	0.13897 (11)	0.0121 (5)	
H15	0.8521	−0.1181	0.1157	0.014*	
O15	0.89286 (8)	0.0599 (2)	0.18944 (7)	0.0107 (3)	
O12	0.94950 (8)	−0.1441 (2)	0.29873 (8)	0.0133 (4)	
H12A	0.9670	−0.2405	0.3221	0.020*	
O13	0.92191 (9)	−0.5475 (3)	0.24839 (8)	0.0174 (4)	
H13A	0.9233	−0.6427	0.2217	0.026*	
O14	0.93366 (8)	−0.4346 (3)	0.11879 (8)	0.0155 (4)	
H14A	0.9728	−0.4926	0.1230	0.023*	
C16	0.94325 (14)	0.0139 (4)	0.09399 (12)	0.0192 (5)	
H16A	0.9205	0.1412	0.0771	0.029*	
H16B	0.9485	−0.0801	0.0586	0.029*	
H16C	0.9893	0.0465	0.1169	0.029*	
C21	0.74537 (12)	0.1967 (4)	0.12541 (11)	0.0135 (5)	
H21	0.7951	0.1923	0.1173	0.016*	
C22	0.74289 (11)	0.1369 (3)	0.19460 (11)	0.0109 (5)	
H22	0.7628	0.2513	0.2231	0.013*	
O22	0.78200 (8)	−0.0481 (2)	0.21047 (7)	0.0106 (3)	
C23	0.66745 (11)	0.0994 (3)	0.20516 (11)	0.0105 (5)	
H23	0.6417	0.2329	0.1986	0.013*	
C24	0.63267 (11)	−0.0554 (4)	0.15733 (11)	0.0120 (5)	
H24	0.6584	−0.1893	0.1615	0.014*	
C25	0.63456 (12)	0.0347 (4)	0.09175 (11)	0.0147 (5)	
H25	0.6115	0.1730	0.0896	0.018*	
O23	0.66298 (8)	0.0302 (2)	0.26878 (7)	0.0111 (3)	
O24	0.56178 (8)	−0.0859 (3)	0.16682 (8)	0.0151 (4)	
H24A	0.5600	−0.1706	0.1966	0.023*	
O25	0.70645 (8)	0.0599 (3)	0.08206 (8)	0.0150 (4)	
C26	0.59920 (13)	−0.0981 (5)	0.03875 (12)	0.0228 (6)	
H26A	0.6034	−0.0334	−0.0024	0.034*	
H26B	0.5498	−0.1132	0.0436	0.034*	
H26C	0.6213	−0.2342	0.0406	0.034*	
O27	0.72203 (9)	0.4015 (2)	0.11823 (8)	0.0160 (4)	
C27	0.74020 (15)	0.4932 (5)	0.06129 (14)	0.0277 (6)	
H27A	0.7895	0.4660	0.0581	0.042*	
H27B	0.7324	0.6423	0.0625	0.042*	
H27C	0.7112	0.4341	0.0242	0.042*	
C31	0.62082 (12)	0.1575 (4)	0.30105 (11)	0.0110 (5)	
H31	0.5776	0.1923	0.2715	0.013*	
C32	0.60030 (11)	0.0409 (4)	0.35762 (10)	0.0110 (5)	
H32	0.5808	−0.0956	0.3422	0.013*	
C33	0.66440 (12)	0.0023 (3)	0.40588 (11)	0.0106 (5)	
H33	0.6966	−0.0894	0.3854	0.013*	
C34	0.70281 (12)	0.2040 (3)	0.42416 (11)	0.0108 (5)	
H34	0.6719	0.2960	0.4460	0.013*	
C35	0.72015 (12)	0.3104 (3)	0.36380 (11)	0.0111 (5)	
H35	0.7515	0.2194	0.3422	0.013*	
O35	0.65577 (8)	0.3437 (2)	0.32144 (7)	0.0117 (3)	
O32	0.54699 (8)	0.1518 (3)	0.38395 (8)	0.0126 (3)	
H32A	0.5645	0.2558	0.4037	0.019*	
O33	0.64458 (8)	−0.1032 (2)	0.45946 (8)	0.0133 (4)	
H33A	0.6805	−0.1388	0.4838	0.020*	
O34	0.76396 (8)	0.1594 (3)	0.46677 (8)	0.0157 (4)	
H34A	0.7898	0.0805	0.4488	0.024*	
C36	0.75423 (13)	0.5176 (4)	0.37637 (12)	0.0187 (5)	
H36A	0.7546	0.5910	0.3359	0.028*	
H36B	0.8023	0.4985	0.3969	0.028*	
H36C	0.7281	0.5980	0.4044	0.028*	

### Atomic displacement parameters (Å^2^)

**Table:**

	*U*^11^	*U*^22^	*U*^33^	*U*^12^	*U*^13^	*U*^23^
OW1	0.0248 (10)	0.0236 (10)	0.0246 (10)	0.0013 (8)	0.0114 (8)	−0.0030 (8)
OW2	0.0130 (9)	0.0193 (9)	0.0394 (12)	−0.0022 (8)	−0.0019 (8)	0.0144 (8)
OW3	0.0211 (10)	0.0208 (9)	0.0218 (10)	−0.0004 (8)	0.0019 (8)	−0.0010 (8)
OW4	0.0354 (12)	0.0274 (11)	0.0349 (12)	−0.0050 (10)	0.0102 (10)	0.0050 (9)
OW5	0.0270 (11)	0.0176 (9)	0.0343 (11)	−0.0025 (8)	−0.0046 (9)	0.0022 (9)
C11	0.0078 (11)	0.0104 (10)	0.0138 (11)	−0.0012 (9)	−0.0008 (9)	−0.0032 (9)
C12	0.0066 (11)	0.0135 (11)	0.0143 (12)	−0.0022 (9)	0.0000 (9)	−0.0007 (9)
C13	0.0112 (11)	0.0085 (10)	0.0171 (12)	−0.0003 (9)	−0.0018 (9)	0.0011 (9)
C14	0.0086 (11)	0.0113 (10)	0.0139 (11)	0.0015 (9)	−0.0025 (9)	−0.0015 (9)
C15	0.0108 (11)	0.0108 (10)	0.0140 (12)	−0.0002 (9)	−0.0006 (9)	−0.0005 (9)
O15	0.0098 (8)	0.0089 (7)	0.0133 (8)	0.0001 (6)	0.0020 (6)	−0.0003 (7)
O12	0.0097 (8)	0.0133 (8)	0.0149 (9)	0.0010 (7)	−0.0055 (6)	0.0006 (7)
O13	0.0251 (9)	0.0082 (8)	0.0185 (9)	0.0040 (7)	0.0017 (7)	0.0012 (7)
O14	0.0131 (8)	0.0163 (8)	0.0164 (9)	0.0063 (7)	−0.0006 (7)	−0.0047 (7)
C16	0.0241 (14)	0.0176 (12)	0.0172 (13)	0.0002 (10)	0.0075 (10)	0.0026 (10)
C21	0.0104 (12)	0.0126 (11)	0.0179 (12)	0.0031 (9)	0.0032 (9)	0.0022 (10)
C22	0.0080 (11)	0.0083 (10)	0.0161 (12)	0.0034 (9)	0.0009 (9)	−0.0015 (9)
O22	0.0055 (8)	0.0099 (7)	0.0156 (8)	0.0017 (6)	−0.0012 (6)	0.0010 (6)
C23	0.0092 (11)	0.0120 (11)	0.0100 (11)	0.0023 (9)	0.0003 (8)	0.0011 (9)
C24	0.0054 (10)	0.0154 (11)	0.0154 (11)	0.0028 (9)	0.0014 (9)	−0.0010 (10)
C25	0.0100 (11)	0.0196 (12)	0.0139 (12)	0.0027 (10)	−0.0010 (9)	0.0000 (10)
O23	0.0088 (8)	0.0131 (8)	0.0113 (8)	0.0038 (6)	0.0016 (6)	0.0013 (7)
O24	0.0084 (8)	0.0199 (9)	0.0167 (9)	−0.0017 (7)	0.0007 (6)	0.0043 (7)
O25	0.0124 (8)	0.0204 (9)	0.0123 (8)	0.0013 (7)	0.0018 (6)	−0.0015 (7)
C26	0.0146 (13)	0.0365 (15)	0.0163 (13)	−0.0014 (11)	−0.0014 (10)	−0.0060 (12)
O27	0.0161 (9)	0.0126 (8)	0.0199 (9)	0.0052 (7)	0.0050 (7)	0.0070 (7)
C27	0.0283 (15)	0.0270 (14)	0.0291 (15)	0.0056 (12)	0.0082 (12)	0.0162 (12)
C31	0.0073 (10)	0.0126 (10)	0.0128 (11)	0.0009 (9)	−0.0004 (8)	−0.0017 (9)
C32	0.0092 (11)	0.0102 (10)	0.0131 (11)	−0.0006 (9)	−0.0004 (9)	−0.0008 (9)
C33	0.0118 (11)	0.0079 (10)	0.0124 (11)	0.0010 (9)	0.0030 (9)	0.0017 (9)
C34	0.0101 (11)	0.0097 (10)	0.0123 (11)	0.0004 (9)	−0.0003 (9)	−0.0017 (9)
C35	0.0074 (11)	0.0118 (11)	0.0137 (12)	0.0013 (9)	−0.0005 (9)	−0.0012 (9)
O35	0.0111 (8)	0.0106 (7)	0.0125 (8)	0.0002 (6)	−0.0015 (6)	0.0001 (6)
O32	0.0087 (8)	0.0153 (8)	0.0142 (8)	0.0009 (7)	0.0029 (6)	0.0009 (7)
O33	0.0105 (8)	0.0141 (8)	0.0142 (8)	0.0013 (7)	−0.0024 (6)	0.0036 (7)
O34	0.0103 (8)	0.0193 (9)	0.0165 (9)	0.0017 (7)	−0.0022 (7)	0.0008 (7)
C36	0.0204 (13)	0.0152 (12)	0.0201 (13)	−0.0065 (10)	0.0006 (10)	−0.0002 (10)

### Geometric parameters (Å, º)

**Table:**

OW1—H101	0.875 (15)	C23—O23	1.432 (3)
OW1—H102	0.879 (15)	C23—C24	1.517 (3)
OW2—H201	0.871 (15)	C23—H23	1.0000
OW2—H202	0.869 (15)	C24—O24	1.426 (3)
OW3—H301	0.878 (14)	C24—C25	1.510 (3)
OW3—H302	0.877 (14)	C24—H24	1.0000
OW4—H401	0.883 (15)	C25—O25	1.442 (3)
OW4—H402	0.883 (15)	C25—C26	1.504 (3)
OW5—H501	0.867 (15)	C25—H25	1.0000
OW5—H502	0.873 (14)	O23—C31	1.400 (3)
C11—O15	1.409 (3)	O24—H24A	0.8400
C11—O22	1.412 (3)	C26—H26A	0.9800
C11—C12	1.519 (3)	C26—H26B	0.9800
C11—H11	1.0000	C26—H26C	0.9800
C12—O12	1.429 (3)	O27—C27	1.428 (3)
C12—C13	1.519 (3)	C27—H27A	0.9800
C12—H12	1.0000	C27—H27B	0.9800
C13—O13	1.435 (3)	C27—H27C	0.9800
C13—C14	1.507 (3)	C31—O35	1.423 (3)
C13—H13	1.0000	C31—C32	1.512 (3)
C14—O14	1.417 (3)	C31—H31	1.0000
C14—C15	1.532 (3)	C32—O32	1.429 (3)
C14—H14	1.0000	C32—C33	1.519 (3)
C15—O15	1.451 (3)	C32—H32	1.0000
C15—C16	1.507 (3)	C33—O33	1.419 (3)
C15—H15	1.0000	C33—C34	1.529 (3)
O12—H12A	0.8400	C33—H33	1.0000
O13—H13A	0.8400	C34—O34	1.419 (3)
O14—H14A	0.8400	C34—C35	1.527 (3)
C16—H16A	0.9800	C34—H34	1.0000
C16—H16B	0.9800	C35—O35	1.451 (3)
C16—H16C	0.9800	C35—C36	1.505 (3)
C21—O27	1.405 (3)	C35—H35	1.0000
C21—O25	1.419 (3)	O32—H32A	0.8400
C21—C22	1.521 (3)	O33—H33A	0.8400
C21—H21	1.0000	O34—H34A	0.8400
C22—O22	1.434 (3)	C36—H36A	0.9800
C22—C23	1.525 (3)	C36—H36B	0.9800
C22—H22	1.0000	C36—H36C	0.9800
			
H101—OW1—H102	106 (2)	C25—C24—C23	107.07 (19)
H201—OW2—H202	109 (2)	O24—C24—H24	110.2
H301—OW3—H302	107 (2)	C25—C24—H24	110.2
H401—OW4—H402	100 (2)	C23—C24—H24	110.2
H501—OW5—H502	111 (2)	O25—C25—C26	108.12 (19)
O15—C11—O22	113.08 (18)	O25—C25—C24	108.40 (17)
O15—C11—C12	110.89 (18)	C26—C25—C24	113.5 (2)
O22—C11—C12	108.63 (17)	O25—C25—H25	108.9
O15—C11—H11	108.0	C26—C25—H25	108.9
O22—C11—H11	108.0	C24—C25—H25	108.9
C12—C11—H11	108.0	C31—O23—C23	112.64 (17)
O12—C12—C13	111.41 (18)	C24—O24—H24A	109.5
O12—C12—C11	104.15 (17)	C21—O25—C25	114.75 (17)
C13—C12—C11	109.73 (18)	C25—C26—H26A	109.5
O12—C12—H12	110.5	C25—C26—H26B	109.5
C13—C12—H12	110.5	H26A—C26—H26B	109.5
C11—C12—H12	110.5	C25—C26—H26C	109.5
O13—C13—C14	110.90 (19)	H26A—C26—H26C	109.5
O13—C13—C12	108.14 (18)	H26B—C26—H26C	109.5
C14—C13—C12	109.94 (19)	C21—O27—C27	111.92 (19)
O13—C13—H13	109.3	O27—C27—H27A	109.5
C14—C13—H13	109.3	O27—C27—H27B	109.5
C12—C13—H13	109.3	H27A—C27—H27B	109.5
O14—C14—C13	109.53 (18)	O27—C27—H27C	109.5
O14—C14—C15	109.00 (18)	H27A—C27—H27C	109.5
C13—C14—C15	109.94 (19)	H27B—C27—H27C	109.5
O14—C14—H14	109.5	O23—C31—O35	111.35 (18)
C13—C14—H14	109.5	O23—C31—C32	108.77 (18)
C15—C14—H14	109.5	O35—C31—C32	110.42 (18)
O15—C15—C16	106.72 (19)	O23—C31—H31	108.7
O15—C15—C14	109.51 (17)	O35—C31—H31	108.7
C16—C15—C14	112.6 (2)	C32—C31—H31	108.7
O15—C15—H15	109.3	O32—C32—C31	109.59 (18)
C16—C15—H15	109.3	O32—C32—C33	112.85 (18)
C14—C15—H15	109.3	C31—C32—C33	109.72 (18)
C11—O15—C15	114.07 (17)	O32—C32—H32	108.2
C12—O12—H12A	109.5	C31—C32—H32	108.2
C13—O13—H13A	109.5	C33—C32—H32	108.2
C14—O14—H14A	109.5	O33—C33—C32	109.45 (18)
C15—C16—H16A	109.5	O33—C33—C34	112.62 (18)
C15—C16—H16B	109.5	C32—C33—C34	110.77 (18)
H16A—C16—H16B	109.5	O33—C33—H33	107.9
C15—C16—H16C	109.5	C32—C33—H33	107.9
H16A—C16—H16C	109.5	C34—C33—H33	107.9
H16B—C16—H16C	109.5	O34—C34—C35	111.48 (18)
O27—C21—O25	112.73 (18)	O34—C34—C33	108.79 (18)
O27—C21—C22	107.14 (19)	C35—C34—C33	109.19 (17)
O25—C21—C22	112.34 (19)	O34—C34—H34	109.1
O27—C21—H21	108.2	C35—C34—H34	109.1
O25—C21—H21	108.2	C33—C34—H34	109.1
C22—C21—H21	108.2	O35—C35—C36	107.24 (18)
O22—C22—C21	110.84 (18)	O35—C35—C34	108.53 (18)
O22—C22—C23	108.53 (17)	C36—C35—C34	113.29 (19)
C21—C22—C23	109.54 (18)	O35—C35—H35	109.2
O22—C22—H22	109.3	C36—C35—H35	109.2
C21—C22—H22	109.3	C34—C35—H35	109.2
C23—C22—H22	109.3	C31—O35—C35	113.29 (16)
C11—O22—C22	113.20 (16)	C32—O32—H32A	109.5
O23—C23—C24	110.01 (18)	C33—O33—H33A	109.5
O23—C23—C22	111.45 (17)	C34—O34—H34A	109.5
C24—C23—C22	110.88 (18)	C35—C36—H36A	109.5
O23—C23—H23	108.1	C35—C36—H36B	109.5
C24—C23—H23	108.1	H36A—C36—H36B	109.5
C22—C23—H23	108.1	C35—C36—H36C	109.5
O24—C24—C25	108.90 (17)	H36A—C36—H36C	109.5
O24—C24—C23	110.37 (18)	H36B—C36—H36C	109.5
			
O15—C11—C12—O12	63.1 (2)	C22—C23—C24—C25	−60.4 (2)
O22—C11—C12—O12	−172.08 (17)	O24—C24—C25—O25	−178.52 (18)
O15—C11—C12—C13	−56.3 (2)	C23—C24—C25—O25	62.1 (2)
O22—C11—C12—C13	68.6 (2)	O24—C24—C25—C26	−58.4 (3)
O12—C12—C13—O13	62.0 (2)	C23—C24—C25—C26	−177.72 (19)
C11—C12—C13—O13	176.82 (17)	C24—C23—O23—C31	−113.6 (2)
O12—C12—C13—C14	−59.2 (2)	C22—C23—O23—C31	122.95 (19)
C11—C12—C13—C14	55.6 (2)	O27—C21—O25—C25	−65.9 (2)
O13—C13—C14—O14	64.7 (2)	C22—C21—O25—C25	55.2 (2)
C12—C13—C14—O14	−175.75 (17)	C26—C25—O25—C21	174.83 (19)
O13—C13—C14—C15	−175.54 (17)	C24—C25—O25—C21	−61.7 (2)
C12—C13—C14—C15	−56.0 (2)	O25—C21—O27—C27	−72.3 (2)
O14—C14—C15—O15	176.01 (17)	C22—C21—O27—C27	163.6 (2)
C13—C14—C15—O15	55.9 (2)	C23—O23—C31—O35	−76.1 (2)
O14—C14—C15—C16	−65.4 (2)	C23—O23—C31—C32	162.03 (16)
C13—C14—C15—C16	174.51 (19)	O23—C31—C32—O32	−169.04 (17)
O22—C11—O15—C15	−63.2 (2)	O35—C31—C32—O32	68.5 (2)
C12—C11—O15—C15	59.1 (2)	O23—C31—C32—C33	66.5 (2)
C16—C15—O15—C11	179.25 (18)	O35—C31—C32—C33	−55.9 (2)
C14—C15—O15—C11	−58.6 (2)	O32—C32—C33—O33	56.2 (2)
O27—C21—C22—O22	−165.21 (16)	C31—C32—C33—O33	178.75 (18)
O25—C21—C22—O22	70.5 (2)	O32—C32—C33—C34	−68.5 (2)
O27—C21—C22—C23	75.1 (2)	C31—C32—C33—C34	54.0 (2)
O25—C21—C22—C23	−49.3 (2)	O33—C33—C34—O34	59.9 (2)
O15—C11—O22—C22	−72.0 (2)	C32—C33—C34—O34	−177.17 (18)
C12—C11—O22—C22	164.44 (18)	O33—C33—C34—C35	−178.26 (18)
C21—C22—O22—C11	91.8 (2)	C32—C33—C34—C35	−55.3 (2)
C23—C22—O22—C11	−147.87 (18)	O34—C34—C35—O35	177.68 (17)
O22—C22—C23—O23	55.3 (2)	C33—C34—C35—O35	57.5 (2)
C21—C22—C23—O23	176.42 (18)	O34—C34—C35—C36	−63.3 (2)
O22—C22—C23—C24	−67.6 (2)	C33—C34—C35—C36	176.44 (19)
C21—C22—C23—C24	53.5 (2)	O23—C31—O35—C35	−59.2 (2)
O23—C23—C24—O24	57.5 (2)	C32—C31—O35—C35	61.8 (2)
C22—C23—C24—O24	−178.80 (17)	C36—C35—O35—C31	174.92 (19)
O23—C23—C24—C25	175.84 (17)	C34—C35—O35—C31	−62.4 (2)

### Hydrogen-bond geometry (Å, º)

**Table:**

*D*—H···*A*	*D*—H	H···*A*	*D*···*A*	*D*—H···*A*
O*W*1—H101···O33^i^	0.88 (3)	1.85 (3)	2.726 (2)	176 (2)
O*W*1—H102···O*W*3^ii^	0.88 (3)	1.95 (3)	2.802 (2)	162 (2)
O*W*1—H102···O32^iii^	0.88 (3)	2.55 (3)	2.976 (2)	110 (2)
O*W*2—H201···O12^iv^	0.88 (3)	2.03 (3)	2.875 (2)	163 (2)
O*W*2—H202···O35^ii^	0.87 (3)	2.08 (3)	2.877 (2)	153 (2)
O*W*3—H301···O*W*5	0.88 (3)	2.04 (3)	2.845 (2)	151 (2)
O*W*3—H302···O13^v^	0.88 (3)	1.96 (3)	2.836 (2)	176 (2)
O*W*4—H401···O*W*3	0.88 (3)	1.97 (3)	2.840 (2)	168 (2)
O*W*4—H402···O*W*1	0.88 (3)	1.92 (3)	2.771 (2)	160 (2)
O*W*5—H501···O33^vi^	0.87 (3)	2.07 (3)	2.918 (2)	168 (2)
O*W*5—H502···O*W*5^vii^	0.87 (3)	2.50 (3)	3.333 (2)	159 (2)
O12—H12*A*···O32^iii^	0.84	2.01	2.767 (2)	149
O13—H13*A*···O15^ii^	0.84	2.10	2.858 (2)	149
O14—H14*A*···O24^iii^	0.84	1.95	2.733 (2)	157
O24—H24*A*···O*W*2	0.84	1.88	2.722 (2)	176
O32—H32*A*···O*W*5^viii^	0.84	2.13	2.864 (2)	146
O33—H33*A*···O34^i^	0.84	1.91	2.684 (2)	152
O34—H34*A*···O*W*4	0.84	1.86	2.687 (2)	168

Symmetry codes: (i) −*x*+3/2, *y*−1/2, −*z*+1; (ii) *x*, *y*−1, *z*; (iii) *x*+1/2, *y*−1/2, *z*; (iv) *x*−1/2, *y*−1/2, *z*; (v) *x*, *y*+1, *z*; (vi) *x*+1/2, *y*+1/2, *z*; (vii) −*x*+2, *y*, −*z*+1; (viii) *x*−1/2, *y*+1/2, *z*.
